# The increased susceptibility to airway infections after preterm birth does not persist into adolescence

**DOI:** 10.1371/journal.pone.0238382

**Published:** 2020-09-03

**Authors:** Anne Louise de Barros Garioud, Frederikke Høeg Skoven, Rasmus Gregersen, Theis Lange, Fsrederik Buchvald, Gorm Greisen

**Affiliations:** 1 Department of Neonatology, Copenhagen University Hospital Rigshospitalet, Copenhagen, Denmark; 2 Section of Biostatistics, University of Copenhagen, Copenhagen, Denmark; 3 Department of Pediatrics, Pulmonary Service, Copenhagen University Hospital Rigshospitalet, Copenhagen, Denmark; Center of Pediatrics, GERMANY

## Abstract

**Introduction:**

Preterm birth is associated with increased risk of airway infections in childhood, more frequent purchase of prescription antibiotics and hospital admissions for airway infections. With this study, we aimed to investigate whether this association persists into adolescence.

**Methods:**

We conducted a longitudinal observational register-based study of a national cohort of all infants born in Denmark in 1992–2007. We used purchase of antibiotics, including penicillins and macrolides, and hospital admissions as proxies for milder and more severe forms of airway infections respectively in 1995–2010. Associations between gestational age (GA), age, year and repeated cross-sectional evaluations of antibiotic purchase and hospital admissions were explored by logistic regression analyses.

**Results:**

We included 1,043,411 children in our study population. We found a statistically significant association between GA and prescription of antibiotics as well as hospital admissions due to airway infections. In the youngest age groups, children with higher GA had lower adjusted mean rates of prescribed antibiotics for airway infections, but from the age of 10–11 years the opposite trend was noted in what appears to be a dose-response relationship. During childhood, we found an inverse dose-response relationship where ex-preterms with GA 23–27 at age 4–5 years had twice the odds of hospital admission compared to children in the same age group born at term. During adolescence, these higher odds diminished and appeared equivalent among teenagers born at term and preterm. We only found statistically significant interactions between gestational age and age.

**Conclusion:**

We confirmed that preterm birth is associated with higher rates of prescribed antibiotics and higher odds of hospitalization for airway infections during childhood. However, in adolescence we found that there was no increase in hospital admissions for airway infections among ex-preterms, whereas adolescents with low GA actually appeared to purchase less prescribed antibiotics. Whether this trend persists into adulthood and the physiological explanation therefor remains to be investigated.

## Introduction

In Denmark about 6.5% of all labors are preterm [1]. Children born preterm have increased susceptibility to infections including respiratory tract infections after discharge, a susceptibility that persists into childhood [2]. Most prematurely born infants need prolonged hospitalization after birth, where the sterile neonatal gastrointestinal tract is colonized by environmental microorganisms. This colonization plays an important role in the development of the early immune system [3]. Preterm infants are exposed to maternal flora but also hospital-based flora through tubes and catheters. Hospital environment bacteria are affected by antibiotic selective pressure, which may alter the establishment of a diverse, healthy microbiome in the newborn, thus increasing the risk of invasive disease [4]. Perinatal infection itself might also influence immune programming [5]. In addition to that, many extremely preterm infants develop some degree of bronchopulmonary dysplasia (BPD). BPD is known to be associated with risks of lower respiratory tract infections such as bronchitis, croup, and pneumonia later in life [6].

Studies on hospital admissions have found higher rates of admission due to respiratory tract infection in children born preterm, but these have primarily targeted cohorts during childhood [5, 7, 8] or included all types of infections [9, 10], thus suggesting a higher disease burden in childhood. Only one study found that the increased risk of infections in childhood persists into adolescence [10]. One study has previously investigated the association between preterm birth and prescription of antibiotics. It did not find lower gestational age (GA) to be associated with higher rates of antibiotic prescription during the first year of life in children born with GA < 32 [11]. We previously found that treatment of asthma-like symptoms in childhood following preterm birth did not persist into adulthood [12, 13], and hypothesized that similar a trend might be applicable for airway infections.

Therefore, we aimed to investigate whether the increased risk of airway infections during childhood following preterm birth weakens during adolescence. To this end, we conducted a study of the association between gestational age, age and purchase of antibiotics for airway infections as well as hospitalization for respiratory tract infections.

## Material and methods

### Cohort, data and ethics

We conducted a longitudinal observational register-based study of a national cohort of all infants born in Denmark in 1992–2007. Data were retrieved from a database created through Statistics Denmark in 2012, where data from all social and health entries from the Medical Birth Registry, the National Patient Register, the Register of Education of the Population and the Cause of Death. Register data were unambiguously linked by individual Central Personal Registration (CPR) numbers. In Denmark, a unique CPR number is assigned to each individual at birth and is required at all interactions with social and health services, which allows for a consistent and reliable data linkage. The Danish Civil Registration System, the research potential in the Danish registers and their limitations have previously been described [14]. All data were anonymized prior to researcher access. The anonymized data can be accessed through Statistics Denmark by research environments who have received prior approval from Statistics Denmark. The study of health consequences of preterm birth was approved by the Danish Data Protection Agency and the Danish Health and Medicines Authority (Jr.no. 2007-41-0806).

The study population included all records of births from January 1st, 1992 to December 31st, 2007 and individuals were included for follow-up on January 1st the year after they turned 2 years of age. Follow-up ended at December 31st, 2010. We excluded individuals who were stillborn or who died before 2 years of age, those who had missing data on GA, gender or birth weight, and those who had abnormal birth weight values defined as differing more than +/- 5 standard deviations (SD) from reference mean birth weights [15] in order to minimize the risk of including coding errors in the study population.

### Outcome

Subjects were evaluated yearly from 2 years of age to end of follow-up. We created two outcomes that in combination could represent reliable markers for airway infections:

- Prescribed antibiotics for airway infections
- Hospital admissions for airway infections

These were chosen to represent a surrogate measure of disease burden and to reflect current and past prescription patterns.

Data on all purchases of prescription penicillin and macrolides from all non-hospital pharmacies in 1997–2010 were obtained from the Danish National Prescription Registry. Hospital pharmacy data are not retrievable on an individual basis from the Danish National Prescription Registry. We included all prescriptions from the Anatomical Therapeutic Chemical (ATC) classes J01C (penicillins) and J01FA (macrolides). These were chosen as they are mainly used for treatment of upper and lower respiratory tract infections. A renewed prescription within 14 days was censored. The outcome was analyzed as number of collected prescriptions per year.

Data on hospital admissions from 1995–2010 were obtained from the Danish National Patient Register, where each admission is linked to at least one diagnosis. We included all diagnoses concerning upper or lower respiratory tract infections selected by their corresponding International Classification of Disease (ICD-10). From 1995–2007 all emergency room and admission diagnoses were included, whereas from 2008–2010 only contacts coded as admissions were included. The antibiotics recommended for otitis in Denmark are the same as those for airway infections. Because otitis is a frequent complication to other upper airway infections in small children, these diagnoses can be difficult to separate. Therefore, otitis diagnoses were also included in the hospitalization outcome. All codes are listed in Table 1. Denmark uses a modified, more detailed version of ICD-10 codes than the international ICD-10 [16]. The outcome was analyzed as binary data, i.e. we analyzed whether an individual had been admitted the year of analysis or not. We chose a binary approach to avoid difference in time at risk, due to the very variable lengths of admission.

**Table 1:** Hospital admission diagnoses included to define outcome variable.

Diagnosis	ICD-10 code	n (%)
Tuberculosis of lung, confirmed by sputum microscopy with or without culture	DA15* (A15*)	93 (0.03)
Respiratory tuberculosis, not confirmed bacteriologically or histologically	DA16* (A16*)	151 (0.05)
Pulmonary mycobacterial infection	DA310* (A31.0*)	<10 (0.00)
Whooping cough	DA37* (A37*)	2,672 (0.82)
Scarlet fever	DA389 (A38.9)	733 (0.22)
Varicella pneumonia	DB012 (B01.2)	52 (0.02)
Measles complicated by pneumonia	DB052 (B05.2)	<10 (0.00)
Cytomegaloviral pneumonitis	DB250 (B25.0)	16 (0.00)
Pulmonary candidiasis	DB371 (B37.1)	<10 (0.00)
Invasive pulmonary aspergillosis	DB440 (B44.0)	28 (0.01)
Other pulmonary aspergillosis	DB441* (B44.1*)	<10 (0.00)
Nonsuppurative otitis media	DH65* (H65*)	16,629 (5.08)
Acute suppurative otitis media	DH660* (H66.0*)	2,1851 (6.68)
Suppurative otitis media, unspecified	DH664 (H66.4)	1,230 (0.38)
Otitis media, unspecified	DH669 (H66.9)	9,751 (2.98)
Acute upper respiratory infections	DJ00-DJ06* (J00-J06*)	125,225 (38.27)
Influenza and pneumonia	DJ09-DJ18* (J09-J18*)	81,019 (24.76)
Other acute lower respiratory infections	DJ20-DJ22* (J20-J22*)	64,006 (19.56)
Peritonsillar abscess	DJ369 (J36.9)	1,818 (0.56)
Retropharyngeal and parapharyngeal abscess	DJ390 (J39.0)	129 (0.04)
Other abscess of pharynx	DJ391 (J39.1)	<10 (0.00)
Bronchitis, not specified as acute or chronic	DJ409 (J40.9)	1,705 (0.52)
Acute interstitial pneumonia	DJ841D (J84.1D)	<10 (0.00)
Cryptogenic organizing pneumonia	DJ841F (J84.1F)	<10 (0.00)
Abscess of lung with pneumonia	DJ851 (J85.1)	70 (0.02)
Abscess of lung without pneumonia	DJ852 (J85.2)	21 (0.01)
Total		327,227 (100)

ICD: International Classification of Disease.

Danish ICD-10 codes followed by international ICD-10 codes in parenthesis.

### Exposure variables

#### Gestational age

Expressed in full weeks and modelled as a categorical variable with 4 groups; term being defined as GA≥37, moderately preterm as GA 32–36, very preterm as GA 28–31 and extremely preterm as GA 23–27 weeks respectively.

#### Age

Divided into groups ranging from 2–3 years through 4–5, 6–7, 8–9, 10–11, 12–13, and up to 14–17 years under parametric assumptions. The oldest age group was further divided in two: 14–15 and 16–17 in the prescriptions analyses to evaluate the aspect of puberty and sexually transmitted diseases. We had least data on the oldest age groups as only the earliest birth cohorts reached 14 or older before follow-up.

### Confounding variables and mediators

In order to evaluate any potential calendar effect, calendar years were included into the analyses grouped into 1997–2001, 2002–2006, and 2007–2010 (prescriptions) and 1995–2000, 2001–2005 and 2006–2010 (admissions) under parametric assumptions. Years 1995–1996 were excluded from analyses on antibiotic prescriptions due to notably lower purchases in those years, possibly due to the fact that the Danish National Prescription Registry was established during 1994.

To these variables, we added well-known risk factors for airway infections and prematurity, which were available in the registers. Were included in the multivariate analyses as potential confounding variables: small-for-gestational age (birth weight <-2SD from reference value [15]), sex, older siblings, delivery method (natural birth or caesarean section, but no further subdivision by elective or acute procedure), maternal educational level (International Standard Classification of Education (ISCED) 2011 categories grouped into 3 levels: ISCED 0–2: level 1, ISCED 3–4: level 2 and ISCED 5–8: level 3). We also included the potential mediators represented by neonatal respiratory diagnoses. These were split into two groups: one group representing “acute neonatal respiratory distress” including (ICD-10 codes) respiratory distress syndrome of newborn (P22.0), transient tachypnea of newborn (P22.1), other respiratory distress of newborn (P22.8) and interstitial emphysema originating in the perinatal period (P25.0) and a second group representing a more chronic neonatal respiratory disease with “bronchopulmonary dysplasia” (P27.1). After a quite drastic change in the treatment approach from mechanical ventilation to a more widespread use of Continuous Positive Airway Pressure (CPAP) in Denmark from 1987 and onwards, the BPD diagnosis in the Danish registers is largely thought to represent the”new BPD” [17].

### Statistics

Since both outcomes were evaluated yearly, we employed a repeated measurements approach, i.e. a mixed effects model, with a first-order autoregressive correlation structure. A mixed effect model is used to account for the fact that we observe the same individual several times and that such measurement cannot be independent. Specifically, in a mixed effect model a random term is included in the model formulation which will capture the special individual—but not time-varying—features, which are otherwise not captured in the explanatory variables. The non-random terms (ie. the explanatory variables) can be interpreted as a usual regression. The mixed effect component is merely needed to ensure valid confidence intervals and p-values when using data where the same individual is included several times.

The number of antibiotic prescriptions were analyzed with linear link function (thereby producing changes in means as the effect estimate), while hospital admissions were analyzed with logistic link function (producing odds ratios as effect estimates).

Two models were employed including GA and age as exposures:

1. Model 1: Adjusted for calendar year and the interactions between GA and age and GA and year.
2. Model 2: As model 1 and adjusted for sex, SGA, older siblings, maternal educational level, delivery method, acute neonatal respiratory distress and BPD.

All data management, analyses, and graphic illustrations were carried out in SAS Statistical Software (Version 9.4, SAS Institute, USA).

## Results

### Study population and unadjusted analyses

**Fig 1:**
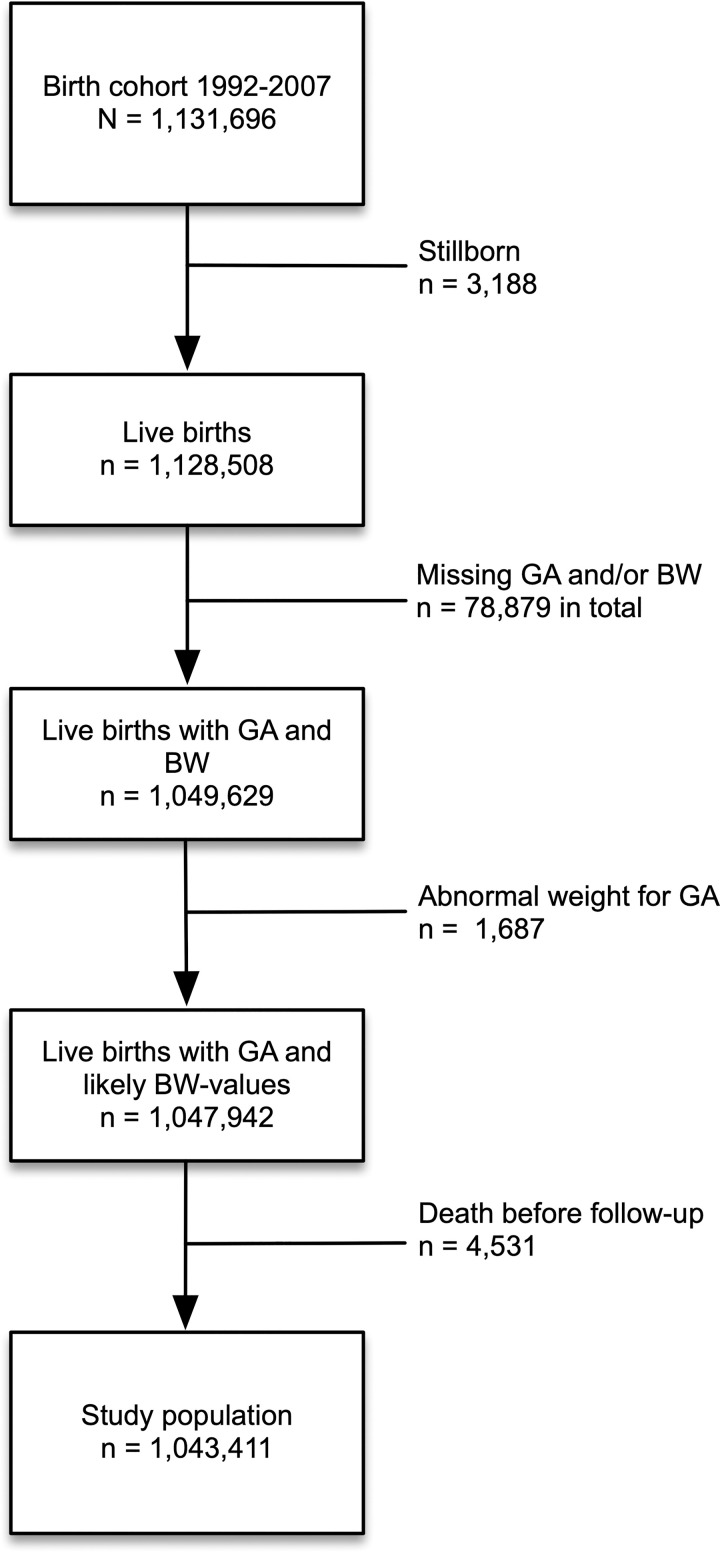
Flow-chart with exclusions from national birth cohort to final study population. GA: gestational age, BW: birth weight.

After exclusions, our study population consisted of 1,043,411 individuals (92% of the birth cohort) born in Denmark between January 1st, 1992 and December 31st, 2007. Fig 1 illustrates the exclusions from birth cohort to our study population.

In the study population, 63,316 (6.05%) of the individuals were born preterm (Table 2). The sex distribution in the total study population was 535,033 males (51.3%) to 508,378 females (48.7%). BPD was primarily seen in the extremely preterm group, whereas acute neonatal respiratory disease was also present in the very preterm and moderately preterm group. Maternal factors such as lower education level and smoking were related to all forms of preterm birth (Table 2).

**Table 2:** Baseline characteristics of final study population in crude numbers (Interquartile Range, IQR, and % respectively).

		GA 37+	GA 32–36	GA 28–31	GA 23–27
		n = 980,095	n = 55,029	n = 6,609	n = 1,678
**Median BW (IQR)**		3,550 (3,220–3,900)	2,500 (2,144–2,861)	1,421 (1,198–1,650)	900 (768–1,033)
**SGA**		23,895 (2.4%)	5,377 (9.8%)	1,340 (20.3%)	229 (13.7%)
**Female gender**		479,358 (48.9%)	25,154 (45.7%)	3,049 (46.1%)	817 (48.7%)
**Caesarean sectio**		145,633 (14.9%)	22,203 (40.4%)	4,503 (68.4%)	685 (58.9%)
**Firstborn**		427,401 (43.7%)	29,680 (54.3%)	3,950 (60.3%)	1,018 (61.2%)
**ISCED-groups**	**0–2 (level 1)**	324,685 (33.3%)	19,467 (35.6%)	2,335 (35.5%)	653 (39.1%)
	**3–4 (level 2)**	373,425 (38.3%)	21,499 (39.3%)	2,612 (39.7%)	634 (37.9%)
	**5–8 (level 3)**	277,074 (28.4%)	13,791 (25.2%)	1,631 (24.8%)	385 (23.0%)
**BPD**		16 (0.0%)	30 (0.1%)	355 (5.4%)	628 (37.4%)
**Acute neonatal respiratory disease**		18,289 (1.9%)	9,666 (17.6%)	4,060 (61.4%)	1,300 (77.5%)

GA: gestational age, BW: birth weight, SGA: small for gestational age, ISCED: International Standard Classification of Education, BPD: bronchopulmonary dysplasia.

Our study yielded a total of 8,951,220 observations of which 203,814 were censored due to emigration or death during the period. Therefore, 8,749,395 observations were analyzed. The number of observations varied among the birth cohorts and calendar years, because the latest birth cohorts had a shorter period of observation.

Our unadjusted data showed a decline in purchase over antibiotic from infancy to early teenage years, with mean yearly number of prescriptions falling gradually from 0.62 to 0.21 from infancy to 13 years of age, after which it showed a small increase to 0.31. Similarly, the proportion of hospitalizations fell from 2.1% in 2-3-year-olds to 0.2 and 0.3% in 12–13 and 14-17-year-olds respectively (Table 3).

**Table 3:** Unadjusted mean number of penicillins and macrolides prescriptions per year and proportion of children with admission for airway infections per year by the study variables.

Variable		Mean number of prescriptions per year (95% CI)	Proportion in % with admission for airway infection per year (95% CI)
Gestational age in weeks	37+	0.38 (0.38–0.38)	0.8 (0.8–0.9)
	32–36	0.43 (0.43–0.44)	1.4 (1.3–1.4)
	28–31	0.49 (0.48–0.50)	2.1 (2.0–2.3)
	23–27	0.55 (0.52–0.57)	3.0 (2.6–3.4)
Age in years	2–3	0.62 (0.62–0.62)	2.1 (2.1–2.2)
	4–5	0.45 (0.45–0.45)	1.0 (0.9–1.0)
	6–7	0.33 (0.33–0.33)	0.5 (0.5–0.5)
	8–9	0.25 (0.25–0.25)	0.3 (0.3–0.3)
	10–11	0.21 (0.20–0.21)	0.2 (0.2–0.2)
	12–13	0.21 (0.21–0.21)	0.2 (0.2–0.2)
	14–17	0.31 (0.31–0.31)	0.3 (0.3–0.4)
Calendar year	97–01 / 95–00*	0.50 (0.50–0.50)	1.6 (1.6–1.7)
	02–06 / 01–05*	0.39 (0.39–0.39)	0.9 (0.9–0.9)
	07–10 / 06–10*	0.33 (0.33–0.33)	0.6 (0.6–0.7)
Bronchopulmonary dysplasia	Yes	0.64 (0.60–0.68)	4.7 (4.0–5.4)
	No	0.39 (0.38–0.39)	0.9 (0.9–0.9)
Acute neonatal respiratory disease	Yes	0.47 (0.46–0.47)	1.8 (1.7–1.8)
	No	0.38 (0.38–0.38)	0.9 (0.9–0.9)
Sex	Female	0.39 (0.39–0.39)	0.7 (0.7–0.7)
	Male	0.38 (0.38–0.38)	1.0 (1.0–1.0)
Small for gestational age	Yes	0.42 (0.42–0.43)	1.2 (1.2–1.3)
	No	0.38 (0.38–0.39)	0.9 (0.9–0.9)
Educational level	ISCED: 0–2	0.40 (0.40–0.40)	1.0 (0.9–1.0)
	ISCED: 3–4	0.39 (0.39–0.39)	0.8 (0.8–0.8)
	ISCED: 5–8	0.36 (0.36–0.36)	0.8 (0.8–0.9)
Mode of delivery	Cesarean section	0.44 (0.43–0.44)	1.2 (1.2–1.2)
	Natural birth	0.38 (0.38–0.38)	0.8 (0.8–0.8)
Older siblings	Firstborn	0.41 (0.41–0.41)	1.0 (0.9–1.0)
	Not firstborn	0.37 (0.37–0.37)	0.8 (0.8–0.8)
Total		0.39 (0.38–0.39)	0.9 (0.9–0.9)

ISCED: International Standard Classification of Education.

The study period was different for the two outcomes. Therefore, the year-groups differ, with the groups marked with (*) being used for the admission outcome.

### Adjusted analyses

#### Purchase of antibiotics for airway infections

**Fig 2:**
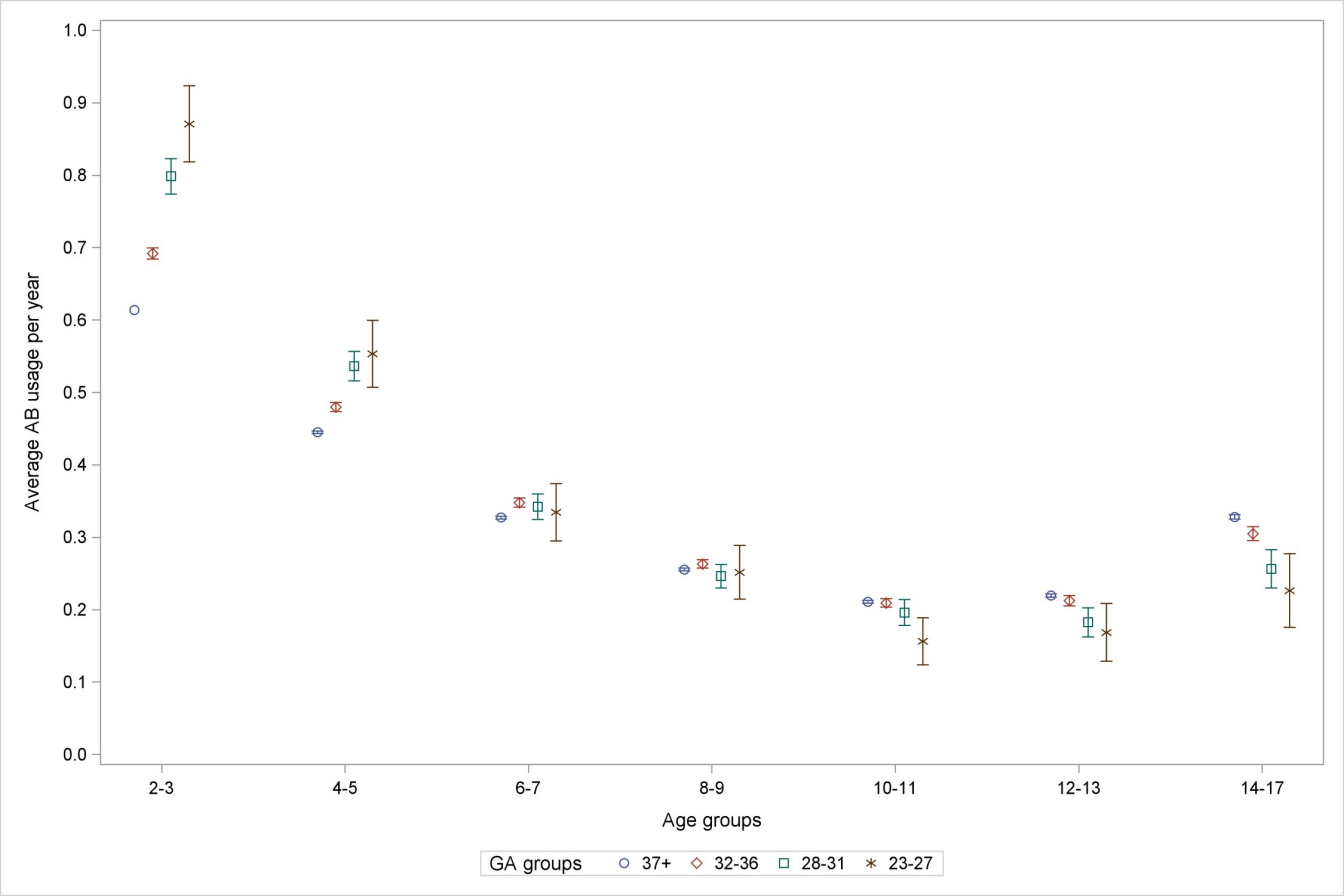
Average antibiotic courses purchased per child per year in GA-groups. Illustrates gradual decrease of the GA-effect up to and including age group 6–7 years, and in older age group an inverse relationship in age groups. The reference group was GA 37+ at age 2–3 year in the years 1997–2001. Adjusted for birth year, sex, SGA, maternal educational level, cesarean section, older sibling, acute respiratory disease and bronchopulmonary dysplasia. GA: gestational age, AB: antibiotics.

**Fig 3:**
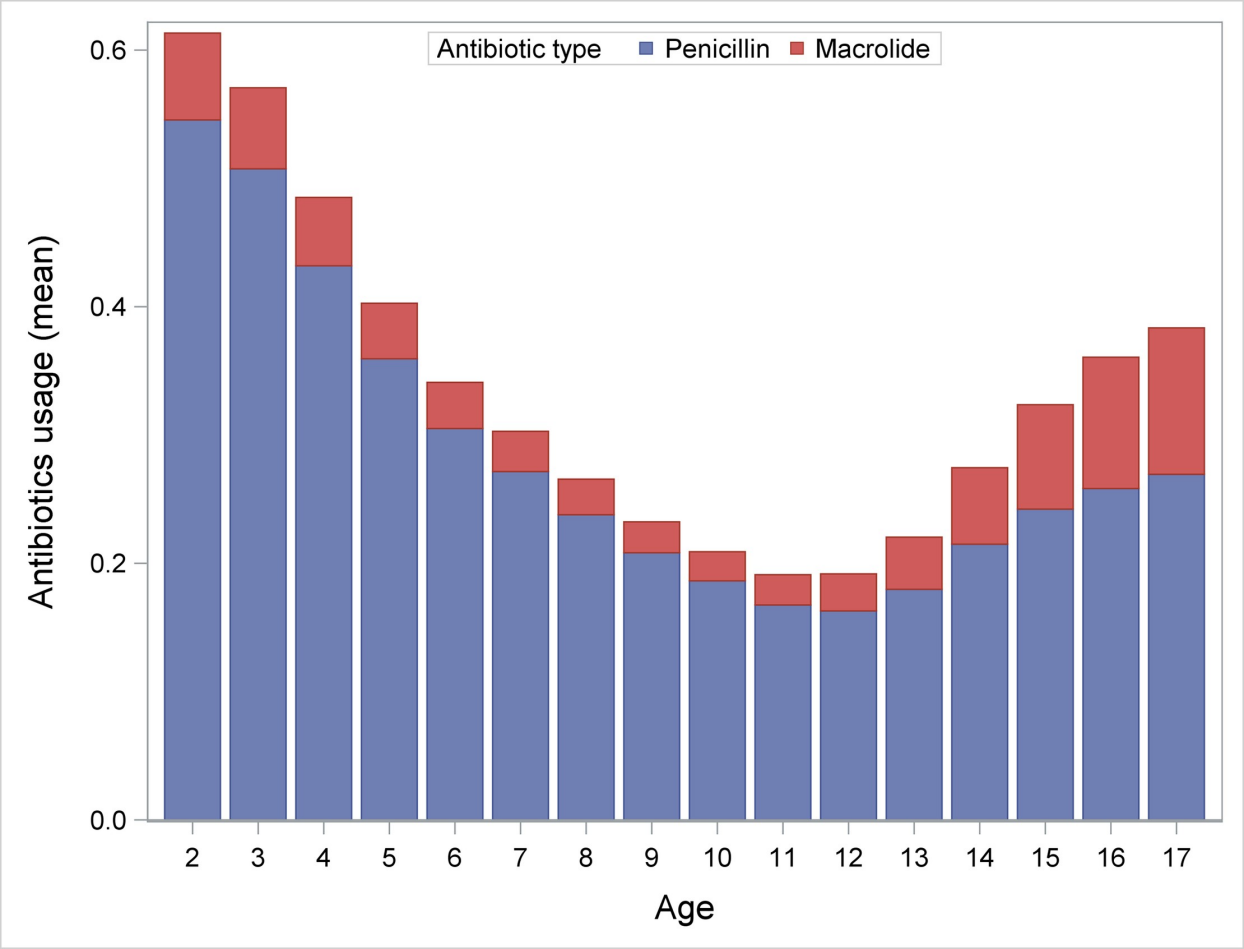
Mean number of purchased antibiotic courses per year classified in types. Macrolide use increased markedly from age 12, but penicillin use also increased somewhat.

The mean number of antibiotic prescriptions redeemed per individual was 0.39 per year. Average number of antibiotic courses purchased per child per year adjusted according to Model 2 are shown in Fig 2. The youngest age group had the highest number of antibiotic purchases for airway infections, and the number of purchases decreased gradually with age until 11–12 years of age, after which it increased again (Fig 3). In the youngest age groups, children with higher GA showed lower adjusted mean rates of purchasing antibiotics for airway infections but above 10–11 years of age, the opposite trend was noted, inversing the dose-response relationship observed in early childhood (Fig 2). We found a slight decrease in antibiotic prescriptions from 1997–2001 to 2002–2007 and further to 2007–2010. Children with neonatal respiratory disease seemed to have an increased use of antibiotics. Likewise, female sex, SGA, being firstborn and caesarean section were also associated with higher usage of antibiotics than the reference group. Maternal education level, ISCED level 3–4 and 5–8 had significant negative mean differences, i.e. higher education level had a small protective effect.

Model 1 and 2 had almost identical mean differences thus it seems that GA is a significant risk factor per se for milder airway infections during childhood, while the mediators and confounders we adjusted for in model 2 comparatively constituted minor risk factors. The full analysis of mean differences in purchase of antibiotics including model 1 and 2 can be found in S1 Table.

In order to explore the surprising prescription patterns in the older age groups, antibiotics were subdivided into penicillins and macrolides. This showed that during childhood the proportion of macrolides was almost stable, whereas from age 11, the overall antibiotic prescription rate increases, with a proportionally bigger increase in the purchase of macrolides (Fig 3).

#### Hospital admissions for airway infections

**Fig 4:**
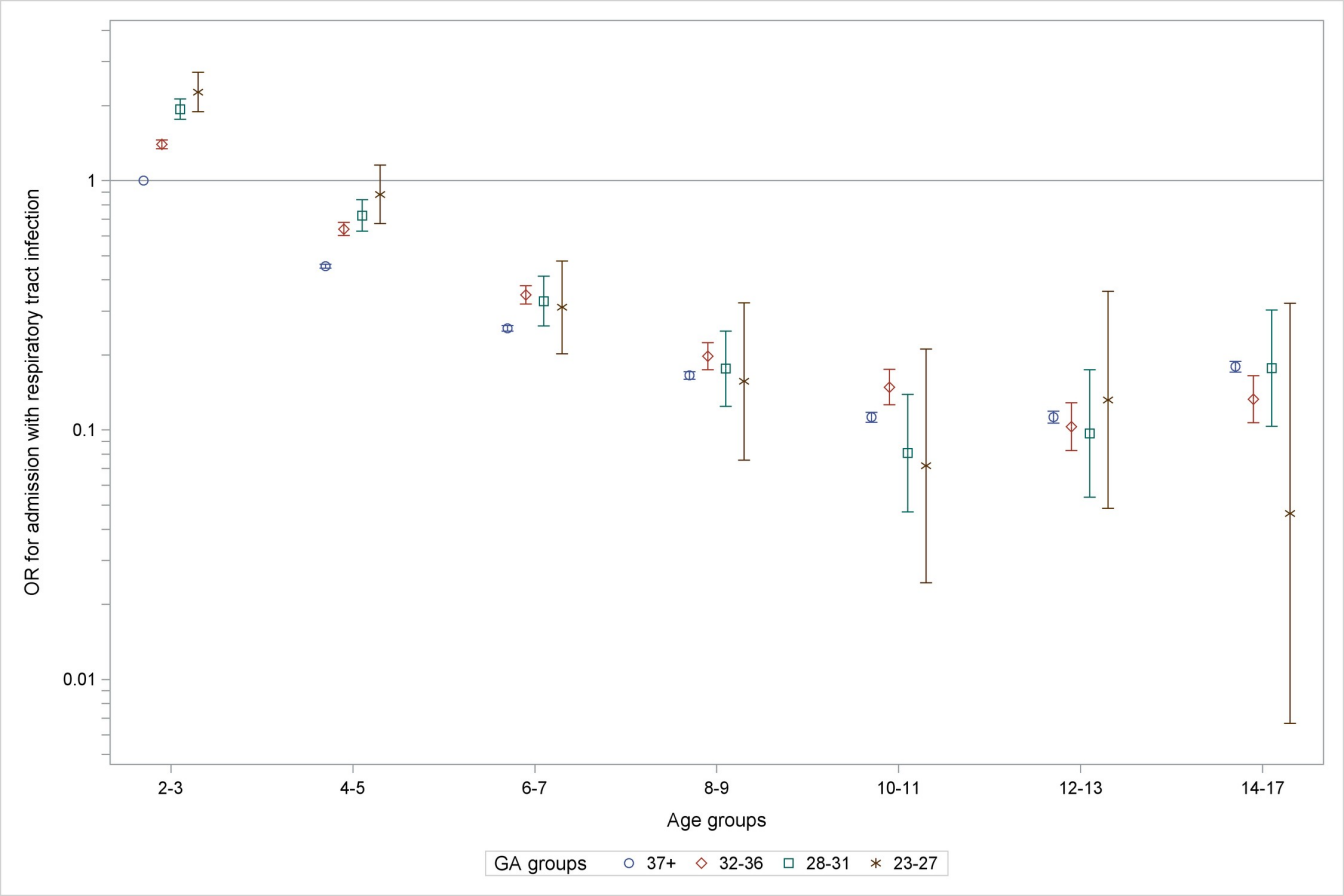
ORs for hospital admissions per GA-group illustrating gradual decrease of the GA-effect. The reference group was GA 37+ at age 2–3. Adjusted for calendar year, sex, SGA, maternal educational level, cesarean section, older sibling, acute respiratory disease and bronchopulmonary disease. OR: odds-ratio, GA: gestational age.

The total number of years with at least one admission for airway infection was 75,229, corresponding to 0.86% of all child-years in the dataset. Table 4 shows odds ratios for hospital admissions adjusted according to Model 1 and 2 respectively. Both models illustrate that in the youngest age group (2–3 years), lower GAs had higher odds of admission for airway infections (Table 4). There was a decline in hospitalizations from infancy to early teenage years: from age group 4–5 years and above, children were less hospitalized than the reference group with ORs under 1, and the ORs gradually decreased with age until the age of 10–11 years, after which there was a small increase (Fig 4). Children born at term had higher odds of admission in the oldest age group. Odds of admission decreased from 1995–2000 to 2001–2005 and again in 2006–2010 with ORs below 1. Neonatal respiratory disease, having no older siblings, caesarean section, and SGA all seemed to increase the odds of hospital admission due to airway infections whereas female sex and higher maternal education levels (ISCED levels 3–4 and 5–8) seemed to lower the odds.

**Table 4:** Odds ratios for admission to hospital.

Variable			Model 1 OR (95% CI)	Model 2 OR (95% CI)
Intercept			0.02 (0.02–0.02)	0.03 (0.03–0.03)
Age	4–5 years		0.46 (0.45–0.46)	0.45 (0.45–0.46)
	6–7 years		0.26 (0.25–0.26)	0.26 (0.25–0.26)
	8–9 years		0.17 (0.16–0.17)	0.17 (0.16–0.17)
	10–11 years		0.11 (0.11–0.12)	0.11 (0.11–0.12)
	12–13 years		0.11 (0.11–0.12)	0.11 (0.11–0.12)
	14–17 years		0.18 (0.17–0.19)	0.18 (0.17–0.19)
Year	01–05		0.91 (0.89–0.93)	0.91 (0.89–0.93)
	06–10		0.86 (0.84–0.88)	0.87 (0.85–0.89)
GA	23–27 weeks		3.64 (2.83–4.69)	2.26 (1.74–2.95)
	28–31 weeks		2.79 (2.44–3.19)	1.94 (1.69–2.23)
	32–36 weeks		1.62 (1.53–1.72)	1.40 (1.32–1.48)
GA*age	23–27 weeks	4–5 years	0.84 (0.65–1.07)	0.85 (0.67–1.09)
	23–27 weeks	6–7 years	0.51 (0.33–0.79)	0.53 (0.34–0.83)
	23–27 weeks	8–9 years	0.38 (0.19–0.79)	0.41 (0.20–0.85)
	23–27 weeks	10–11 years	0.24 (0.08–0.73)	0.27 (0.09–0.82)
	23–27 weeks	12–13 years	0.41 (0.14–1.15)	0.48 (0.17–1.38)
	23–27 weeks	14–17 years	0.08 (0.01–0.57)	0.11 (0.01–0.75)
GA*age	28–31 weeks	4–5 years	0.82 (0.71–0.94)	0.83 (0.71–0.96)
	28–31 weeks	6–7 years	0.65 (0.51–0.83)	0.67 (0.52–0.85)
	28–31 weeks	8–9 years	0.53 (0.37–0.75)	0.55 (0.39–0.79)
	28–31 weeks	10–11 years	0.35 (0.2–0.61)	0.37 (0.21–0.65)
	28–31 weeks	12–13 years	0.41 (0.23–0.75)	0.45 (0.24–0.82)
	28–31 weeks	14–17 years	0.47 (0.27–0.83)	0.51 (0.29–0.90)
GA*age	32–36 weeks	4–5 years	1.01 (0.94–1.08)	1.01 (0.94–1.08)
	32–36 weeks	6–7 years	0.97 (0.88–1.06)	0.98 (0.89–1.07)
	32–36 weeks	8–9 years	0.86 (0.75–0.98)	0.86 (0.75–0.98)
	32–36 weeks	10–11 years	0.93 (0.78–1.11)	0.94 (0.79–1.12)
	32–36 weeks	12–13 years	0.66 (0.52–0.83)	0.65 (0.52–0.82)
	32–36 weeks	14–17 years	0.53 (0.42–0.66)	0.53 (0.42–0.66)
GA*year	23–27 weeks	01–05	1.06 (0.76–1.48)	0.91 (0.77–1.54)
	23–27 weeks	06–10	1.32 (0.94–1.84)	1.09 (0.77–1.54)
	28–31 weeks	01–05	1.05 (0.88–1.29)	1.00 (0.84–1.19)
	28–31 weeks	06–10	1.07 (0.89–1.29)	0.99 (0.82–1.20)
	32–36 weeks	01–05	0.99 (0.92–1.10)	0.99 (0.92–1.07)
	32–36 weeks	06–10	1.01 (0.93–1.10)	1.00 (0.93–1.09)
Female sex				0.70 (0.69–0.71)
SGA				1.22 (1.17–1.28
ISCED		3–4 (level 2)		0.85 (0.84–0.87)
		5–8 (level 3)		0.81 (0.79–0.83)
Caesarian sectio				1.20 (1.17–1.23)
First born				1.14 (1.12–1.16)
Acute neonatal respiratory disease				1.35 (1.29–1.41)
BPD				1.72 (1.39–2.12)

OR: Odds Ratio, GA: gestational age, SGA: small for gestational age, ISCED: International Standard Classification of Education, BPD: bronchopulmonary dysplasia.

The intercept represents the odds for children age 2–3 years, with GA ≥37 weeks, in the years 1995–2000.

## Discussion

In this large longitudinal register study, we confirmed that preterm birth is associated with increased purchase of antibiotics and higher odds of hospitalization for airway infections with a peak effect in infancy, suggesting a susceptibility for airway infections. For the first time, we document that throughout childhood this association diminishes.

Additionally, in the youngest age group we found that the odds of hospital admission due to airway infections were twice as high and the prescription rate of antibiotics was almost 50% higher for those born extremely preterm compared to children born at term. In general, the children had fewest prescriptions and admissions around 10–13 years of age in both ex-preterm and term-born children. During adolescence, the relationship between GA and prescriptions seemed to reverse, while the odds of admissions appeared to even out across GA-groups. Thus, the average antibiotic prescription rate was roughly 1.5 times higher for the term-born children than children born at GA 23–27 in adolescence. A large part of the overall increase in antibiotic prescriptions after 11 years of age consisted of macrolides. Macrolides are commonly used for infections caused by chlamydiae, mycoplasmae but also for streptococcal infections in individuals with penicillin allergy [18]. This increase in use of macrolides could potentially reflect the risk of atypical pneumonia infections which are common in school aged children and young adults [19]. In the same manner, the most common bacterial genital infection, chlamydia trachomatis could cause an increase in adolescents and young adults, and so could the second most common–mycoplasma genitalum [20]. Lastly, streptococcal infections such as erysipelas and wound infections could also contribute to the increase in prescriptions of macrolides which often are favored by clinicians due to fewer daily dosages than ordinary penicillins, thereby resulting in better treatment adherence [18].

There are many potential reasons for the increased prescription rate of antibiotics for young children born preterm. Mothers of children born preterm experience greater emotional distress such as worries about their children, depression, anxiety, and post-traumatic stress than mothers of children born at term. Parents’ view on premature infants could be affected by their prolonged hospitalization after birth. A large longitudinal cohort study found that at 5 years of age, parents were still more likely to rate their child’s health as poorer with decreasing GA [7]. These factors may all lead to more visits to physicians or general practitioners. The frequent visits, parental concern, and the chronic medical complications of prematurity after long hospitalization might also influence the physicians’ perception. This could bias physicians into different antibiotic prescription patterns [11].

More remarkably, the reversed relationship between GA and antibiotic prescriptions during adolescence could be explained by these children remaining socially more protected and thereby less exposed to potential sources of infection in early teenage years. Several studies support this theory; when ex-preterm children reach school age, some tend to have reduced social skills compared with children born at term, and they tend to be less likely to participate in after-school sports activities [21]. An ex-preterm behavioral phenotype has been suggested, characterized by anxiety, attention and social difficulties along with an association with psychiatric disorders [22]. Such factors may lead to social isolation with decreased exposure for infections. However, these are speculations and the actual reasons for the observed reversal in prescriptions of antibiotics during adolescence remains to be investigated.

One other study investigated the relationship between antibiotic prescriptions and prematurity, but where it focused on investigating the first year of life, our study started follow up at the age of two. The study did not find that decreasing GA was associated with higher rates of antibiotic prescriptions in infancy, but it found male sex, BPD and older children in the household to create an increased risk [11]. Thus, our study is, as far as we know, the only one investigating antibiotic prescriptions from early childhood to adolescence, and the first one to combine prescriptions and admissions to investigate the different degrees of severity on the spectrum of airway infections.

Several studies have demonstrated increased risks of hospital admission in children born preterm. Definitions, however, varied from admission for all causes [7], admission for all kinds of infections [9, 10], lower respiratory tract infections [5] and to admission for respiratory infections or asthma, only [8]. Most studies ended follow-up at between 5 and 15 years of age, while one study followed up until the age of 18 [10]. This study concluded that the increased risk of admission persisted into adolescence, while our data suggests that it levels out. They studied admission for all kinds of infection, although two thirds were respiratory. Thus, most likely the reason for the different conclusions lies in limited statistical power.

The biggest strength of this study is its large size, with an almost complete national birth cohort over a significant period of time. We also had access to emigrational data and death both in the country and abroad which contributed to reliable censoring. Our data were routinely collected and linked to a unique central personal registration number (CPR number). These data include important confounders and mediators with very low risk of selection- and recall biases, consistent data linkage with high validity, and low risk of loss of data [16]. The neonatal respiratory diagnoses were included in the analyses as mediators. Although this adds a potential risk for over-adjusting and thereby underestimating the GA-effect, we found it valuable to study the GA “per se” effect. Strictly speaking, the chain of causation starts before preterm birth with the dispositions and exposures which set the scene of preterm birth, over the premature exposure of the immature infant to oxygen, microorganisms and a degree of nutritional failure, to the complications of the neonatal period. Most importantly, we were interested in studying the effect of immaturity at birth (with GA as the simple measure) and the potential change with age in childhood and adolescence, and found that the effect and its diminishment with age was robust to adjustment for confounders and mediators.

The most important weaknesses are that prescription of antibiotics and admissions were used as a proxy for airway infections in milder and more severe forms respectively. However, prescriptions are automatically registered when patients purchase the medication in out-of-hospital pharmacies and digitally reported to the Danish National Prescription Registry, providing high quality data. In-hospital pharmacies are not included in the database and have no sales, therefore only medications delivered at discharge would be missing in the study. We interpreted the patient having redeemed the prescription and bought the medication as a high rate of compliance, which adds to the study’s strengths. However, whether the drug has actually been administrated and used as prescribed is not known. Penicillin is most commonly prescribed in the primary sector for airway infections: bacterial acute otitis media, acute tonsillitis and pneumonia [23]. We cannot rule out that penicillin in some cases could have been prescribed for other local infections such as skin infections. However, we did not have access to primary sector diagnostic codes, making prescription of oral antibiotics the best available marker for milder airway infections. In Denmark, every admission to a hospital must be linked to at least one diagnosis, which is linked to the patient’s CPR number and then reported to Danish National Patient Register. It can be argued that middle ear infections do not belong to the same category of airway infections as lower respiratory tract infections. The diagnoses constituted approximately 15% of all hospitalization for airway infections, whereas other upper respiratory tract infections and lower respiratory tract infections accounted for a little less than 40% and 45% respectively. Otitis being a common complication to upper airway infections in small children due to the edema created in the nasopharynx, the diagnoses can be difficult to keep separate. The risk of a bacterial superinfection would probably depend on the colonization of the upper airways, including the effect of previous antibiotic courses. We argue that if a diagnosis of otitis is severe enough to warrant hospitalization, it would most often be linked to a relevant airway infection, although this cannot be known with certainty in the presented study setting.

Our study design did not allow us to explore a possible treatment bias, i.e. that infants born preterm could be more likely to receive antibiotic medication for viral infections at a lower clinical threshold than children born at term. Denmark has strict national guidelines regarding prescription of antibiotics, although varying adherence to guidelines cannot be excluded.

## Conclusions

In this large longitudinal study, we found that preterm birth is associated with an increase in airway infections with a peak effect in infancy. During childhood the effect of prematurity lessens, and in adolescence it seems to even out across all GA-groups with a potential trend towards fewer antibiotic prescriptions in the ex-premature than in the ex-term. This finding remains to be confirmed and explained in future investigations.

## Supporting information

S1 Table Mean differences for antibiotic prescriptions. (DOCX)
