# Correction: The increased susceptibility to airway infections after preterm birth does not persist into adolescence

**DOI:** 10.1371/journal.pone.0244952

**Published:** 2020-12-31

**Authors:** The PLOS ONE Staff

The fifth author's name is spelled incorrectly. The publisher apologizes for the error. The correct name is: Frederik Buchvald. The correct citation is: Garioud ALdB, Skoven FH, Gregersen R, Lange T, Buchvald F, Greisen G (2020) The increased susceptibility to airway infections after preterm birth does not persist into adolescence. PLoS ONE 15(9): e0238382. https://doi.org/10.1371/journal.pone.0238382
