# Supplementary material for: The increased susceptibility to airway infections after preterm birth does not persist into adolescence
Source: PLoS One. 2020 Sep 3;15(9):e0238382. doi: 10.1371/journal.pone.0238382 (PMC7470365; doi:10.1371/journal.pone.0238382)
Supplement: S1 Table — (DOCX) [file pone.0238382.s001.docx]

**S1 Table. Mean differences for antibiotic prescriptions.**

|  |  |  | Model 1 |  | Model 2 |  |
| --- | --- | --- | --- | --- | --- | --- |
|  |  |  | p-value^a^ | Mean differences (95% CI) | p-value^a^ | Mean differences (95% CI) |
| Intercept |  |  |  | 0.62 (0.62-0.63) |  | 0.61 (0.610.62) |
| Age | 4-5 |  | <0.0001 | -0.168 (-0.17- -0.166) | <0.0001 | -0.169 (-0.17- -0.167) |
|  | 6-7 |  |  | -0.285 (-0.287- -0.283) |  | -0.287 (-0.289- -0.285) |
|  | 8-9 |  |  | -0.357 (-0.358- -0.355) |  | -0.359 (-0.361- -0.357) |
|  | 10-11 |  |  | -0.4 (-0.402- -0.398) |  | -0.403 (-0.406- -0.401) |
|  | 12-13 |  |  | -0.393 (-0.395- -0.39) |  | -0.397 (-0.399- -0.394) |
|  | 14-15 |  |  | -0.302 (-0.305- -0.299) |  | -0.306 (-0.309- -0.303) |
|  | 16-17 |  |  | -0.231 (-0.236- -0.227) |  | -0.236 (-0.24- -0.232) |
| Year | 02-06 |  | <0.0001 | -0.006 (-0.008- -0.004) | <0.0001 | -0.004 (-0.006- -0.002) |
|  | 07-10 |  |  | -0.03 (-0.032- -0.027) |  | -0.026 (-0.028- -0.024) |
| GA | 23-27 |  | <0.0001 | 0.35 (0.28-0.42) | <0.0001 | 0.28 (0.21-0.35) |
|  | 28-31 |  |  | 0.26 (0.23-0.29) |  | 0.21 (0.18-0.24) |
|  | 32-36 |  |  | 0.11 (0.10-0.12) |  | 0.09 (0.08-0.10) |
| GA*age | 23-27 | 4-5 | <0.0001 | -0.157 (-0.209- -0.104) | <0.0001 | -0.148 (-0.201- -0.095) |
|  | 23-27 | 6-7 |  | -0.254 (-0.31- -0.198) |  | -0.254 (-0.302- -0.189) |
|  | 23-27 | 8-9 |  | -0.27 (-0.331- -0.21) |  | -0.252 (-0.313- -0.192) |
|  | 23-27 | 10-11 |  | -0.326 (-0.388- -0.264) |  | -0.301 (-0.363- -0.238) |
|  | 23-27 | 12-13 |  | -0.33 (-0.398- -0.261) |  | -0.294 (-0.363- -0.238) |
|  | 23-27 | 14-15 |  | -0.371 (-0.457- -0.286) |  | -0.325 (-0.411- -0.239) |
|  | 23-27 | 16-17 |  | -0.455 (-0.552- -0.359) |  | -0.398 (-0.496- -0.301) |
| GA*age | 28-31 | 4-5 | <0.0001 | -0.096 (-0.122- -0.07) | <0.0001 | -0.092 (-0.117- -0.066) |
|  | 28-31 | 6-7 |  | -0.166 (-0.193- -0.14) |  | -0.164 (-0.191- -0.137) |
|  | 28-31 | 8-9 |  | -0.187 (-0.216- -0.159) |  | -0.182 (-0.211- -0.154) |
|  | 28-31 | 10-11 |  | -0.193 (-0.224- -0.163) |  | -0.186 (-0.217- -0.155) |
|  | 28-31 | 12-13 |  | -0.216 (-0.249- -0.182) |  | -0.207 (-0.241- -0.174) |
|  | 28-31 | 14-15 |  | -0.248 (-0.29- -0.207) |  | -0.238 (-0.279- -0.196) |
|  | 28-31 | 16-17 |  | -0.263 (-0.317- -0.209) |  | -0.253 (-0.307- -0.199) |
| GA*age | 32-36 | 4-5 | <0.0001 | -0.043 (-0.051- -0.035) | <0.0001 | -0.043 (-0.051- -0.035) |
|  | 32-36 | 6-7 |  | -0.056 (-0.065- -0.047) |  | -0.055 (-0.064- -0.047) |
|  | 32-36 | 8-9 |  | -0.067 (-0.076- -0.058) |  | -0.066 (-0.076- -0.057) |
|  | 32-36 | 10-11 |  | -0.076 (-0.086- -0.066) |  | -0.075 (-0.085- -0.065) |
|  | 32-36 | 12-13 |  | -0.81 (-0.092- -0.07) |  | -0.079 (-0.09- -0.068) |
|  | 32-36 | 14-15 |  | -0.093 (-0.107- -0.079) |  | -0.092 (-0.106- -0.078) |
|  | 32-36 | 16-17 |  | -0.101 (-0.121- -0.82) |  | -0.1 (-0.12- -0.81) |
| GA*year | 23-27 | 02-06 | 0.06 | -0.013 (-0.08-0.05) | 0.01 | -0.031 (-0.099-0.04) |
|  | 23-27 | 07-10 |  | -0.002 (-0.075-0.07) |  | -0.035 (-0.109-0.04) |
|  | 28-31 | 02-06 |  | -0.032 (-0.06- -0.003) |  | -0.037 (-0.066- -0.008) |
|  | 28-31 | 07-10 |  | -0.031 (-0.063-<0.001) |  | -0.04 (-0.072- -0.007) |
|  | 32-36 | 02-06 |  | -0.011 (-0.02- -0.001) |  | -0.012 (-0.021- -0.003) |
|  | 23-27 | 02-06 |  | -0.013 (-0.08-0.05) |  | -0.031 (-0.099-0.04) |
| Female sex | | | NA |  | <0.0001 | 0.01 (0.01- 0.01) |
| SGA | | | NA |  | <0.0001 | 0.02 (0.01-0.02) |
| ISCED | | 3-4 (Level 2) | NA |  | <0.0001 | -0.011 (-0.013- -0.009) |
|  |  | 5-8 (Level 3) | NA |  | <0.0001 | -0.056 (-0.058 - -0.054) |
| Caesarian sectio | | | NA |  | <0.0001 | 0.03 (0.03-0.04) |
| First born | | | NA |  | <0.0001 | 0.04 (0.04-0.04) |
| Acute neonatal respiratory disease | | | NA |  | <0.0001 | 0.03 (0.03-0.04) |
| BPD | | | NA |  | <0.0001 | 0.10 (0.05-0.15) |

The intercept represents the expected number of prescriptions in children age 2-3 years, with GA >37 weeks in the years 1997-2001.

^a^P-values are type-3 test.
